# Case of Excision of a Portion of the Ribs, and Also of the Pleura

**Published:** 1818-10-01

**Authors:** Chevalier Richerand

**Affiliations:** Professor of the Faculty of Medicine, and Chief Surgeon to the Hospital of Saint Louis


### *Case of Excision of a Portion of the Ribs, and also of the Pleura.*

By Chevalier Richerand,

Professor of the Faculty of Medicine, and Chief Surgeon to the Hospital of Saint Louis.

M. MICHELLEAU, a health officer of Nemours, was affected, during three years, with a cancerous tumour over the region of the heart, which was extirpated in the month of January, 1818; but a bleeding fungus was frequently reproduced, notwithstanding the application of cauteries and caustics. He now came to Paris and M. Richerand found an enormous fungus rising from the wound, and discharging a reddish and horribly fœtid sanies. Yet the patient did not suffer much pain. He had neither colliquative sweats, nor diarrhœa; and although troubled with a chronic cough, his age and general state of health presented nothing unfavourable to an operation. It was therefore determined to remove a portion of rib or ribs, if necessary, as the seat of disease was considered to be there. Professor Dupuytren, and other surgeons of distinction, were present, and assisted at this formidable operation.

I commenced (says M. Richerand) by enlarging the wound through the medium of a crucial incision, and discovered the sixth rib enlarged and red for four inches in length. With a bistoury I separated the attachments of the intercostal muscles, above and below, through this space, and then with a small Hey's saw I cut through the rib in two places, and removed the diseased portion, carefully detaching, by means of a spatula, the costal pleura from the internal face of the rib. The seventh rib was now found to be diseased, to an equal extent, and was removed in a similar manner, but with much more difficulty, and not without penetrating the cavity of the chest by a slight rent in the pleura. This membrane itself was now discovered to be in a thickened, diseased state; and, in short, to be the tissue whence the fungous vegetation sprang. It was diseased *eight square inches* in extent! To leave this behind was to leave the operation unfinished, and therefore the whole of it was removed by the scissors. Not a drop of blood was lost. At this moment the air rushed in, the left lung collapsed, and, with the heart enveloped in its pericardium, pressed and presented itself at the wound. The wound was instantly covered with adhesive plaster to prevent suffocation. The anxiety and difficulty of breathing were now extreme, and continued so for twelve hours after the operation. The patient passed the night erect, and without sleep. Towards the morning sinapisms were applied to the soles of the feet, and insides of the thighs, rendering the breathing easier. From this moment the pulse rose and the strength increased. The patient was kept on liquids. Three days passed thus. The fever was moderate, but the oppression of the breath was sufficient to prevent sleep. Ninety-six hours after the operation, we removed the dressings. The pericardium and lung had contracted adhesions round the circumference of the wound, which now formed a kind of window, through which we distinctly saw the action of the heart through its transparent covering. Fortunately, the adhesion of the heart to the lung was not complete, and left a sufficient passage for a copious serous discharge which issued from the wound for ten or twelve days, in the quantity of half a pint a day. On the 13th day this discharge ceased; and by the 18th day, the adhesion between the pericardium and lung was complete, and no air entered from without after that period. The patient could now lie down and sleep; his appetite and strength returned; the wound healed, and he perfectly recovered.

---

REMARKS BY THE EDITORS.

A special Report was made by Deschamps and Percy, on this brilliant operation, and while they plainly discover their jealousy of the progress of surgery in this country, they pretty openly insinuate that they have now got a head of us, and that this operation eclipses those of Abernethy, Cooper, &c. in England. But we beg leave to remark, that the great merit of M. Richerand's operation consisted in the *boldness* of the attempt; for the *difficulty* was trifling. By this we do not mean to detract from the well-earned fame of the operator.

SECOND

